# Effectiveness of perioperative calcium and vitamin D supplementation in preventing post-thyroidectomy hypocalcaemia: network meta-analysis of randomized trials

**DOI:** 10.1093/bjsopen/zrag093

**Published:** 2026-07-03

**Authors:** Vasileios Gkanis, Dimitrios Papaconstantinou, Sophocles Lanitis, Nikolaos Dafnios, Ioannis Papakonstantinou, Konstantinos Nastos

**Affiliations:** Second Surgical Department and Unit of Surgical Oncology, ‘Korgialeneio—Benakeio’ Hellenic Red Cross, General Hospital of Athens, Athens, Greece; Third Department of Surgery, National and Kapodistrian University of Athens (NKUA), Attikon University Hospital, Athens, Greece; Second Surgical Department and Unit of Surgical Oncology, ‘Korgialeneio—Benakeio’ Hellenic Red Cross, General Hospital of Athens, Athens, Greece; Second Department of Surgery, National and Kapodistrian University of Athens (NKUA), Aretaieio Hospital, Athens, Greece; Second Department of Surgery, National and Kapodistrian University of Athens (NKUA), Aretaieio Hospital, Athens, Greece; Third Department of Surgery, National and Kapodistrian University of Athens (NKUA), Attikon University Hospital, Athens, Greece

## Abstract

**Background:**

Post-thyroidectomy hypocalcaemia is the most common complication after total thyroidectomy. Although perioperative calcium and vitamin D supplementation is widely used, the comparative effectiveness of different prophylactic strategies remains uncertain.

**Methods:**

A systematic review and Bayesian network meta-analysis of randomized clinical trials was performed according to PRISMA guidelines. PubMed, Scopus, Web of Science, and the Cochrane Library were searched from inception to 7 May 2026. Adult patients undergoing total or near-total thyroidectomy were included. Three strategies were compared: no routine supplementation, calcium alone, and calcium combined with vitamin D analogues. Primary outcomes were clinical hypocalcaemia, biochemical hypocalcaemia, and need for intravenous calcium supplementation; length of hospital stay was secondary. Random-effects network meta-analysis estimated odds ratios with 95% credible intervals. Risk of bias was assessed using RoB 2. The protocol was registered in PROSPERO (CRD420251068837).

**Results:**

Twenty randomized trials including 3669 patients were analysed. Calcium plus vitamin D significantly reduced clinical hypocalcaemia *versus* no supplementation (odds ratio 0.31, 95% credible interval 0.17 to 0.51) and calcium alone (odds ratio 0.52, 0.25 to 1.04). Combination therapy also reduced biochemical hypocalcaemia compared with no supplementation (odds ratio 0.27, 0.17 to 0.42) and calcium alone (odds ratio 0.44, 0.22 to 0.83), and markedly decreased intravenous calcium requirements (odds ratio 0.15, 0.05 to 0.32). Calcium monotherapy showed no significant benefit. Combination therapy ranked as most effective and was associated with shorter length of hospital stay (mean difference 0.44 days).

**Conclusions:**

Perioperative calcium combined with vitamin D is the most effective strategy for preventing post-thyroidectomy hypocalcaemia and reducing intravenous calcium use, supporting routine postoperative implementation despite heterogeneity in supplementation protocols.

## Teaser

This Bayesian network meta-analysis of 20 randomized trials (3669 patients) compared prophylactic strategies for preventing post-thyroidectomy hypocalcaemia. Calcium combined with vitamin D was the most effective approach, significantly reducing clinical and biochemical hypocalcaemia, as well as the need for intravenous calcium, whereas calcium alone showed no clear benefit. These findings support routine use of combination therapy to standardize postoperative care and facilitate safer recovery pathways.

## Introduction

Total thyroidectomy represents the standard surgical treatment for differentiated thyroid cancer and bilateral benign thyroid disease, with expanding indications including compressive symptoms, Graves’ disease, and indeterminate cytology^1,2^. Although generally safe in high-volume centres, hypocalcaemia—mainly due to transient or permanent hypoparathyroidism—remains the most common postoperative complication, with reported incidence rates of 13–49%^3–6^.

Post-thyroidectomy hypocalcaemia results from parathyroid devascularization or inadvertent excision, leading to reduced parathyroid hormone (PTH) secretion^7^ and decreased serum calcium levels. Clinically, it may present with perioral numbness, paraesthesia, carpopedal spasm, or, in severe cases, tetany, seizures, laryngospasm, and cardiac arrhythmias^8–10^. Most cases are transient or protracted, resolving within weeks to months as parathyroid function recovers^11,12^, whereas a minority persist beyond 6–12 months and are classified as permanent, requiring lifelong supplementation and adversely affecting quality of life^13–15^. Serum calcium levels typically reach their nadir within 24–72 hours after surgery, necessitating close monitoring and often limiting early discharge^16^.

To address this common complication, several prophylactic and therapeutic strategies have been proposed. Current guidelines from the American Thyroid Association^7^, the European Society of Endocrine Surgeons^17^, and the British Association of Endocrine and Thyroid Surgeons recommend risk stratification based on early postoperative calcium and PTH levels to guide selective supplementation^18–21^. However, PTH testing is not universally available, may increase costs, and does not fully predict hypocalcaemia, which can still occur despite initially normal levels^22–24^.

Three principal strategies are currently adopted worldwide: calcium/vitamin D supplementation only in symptomatic patients^25,26^, supplementation guided by early biochemical markers such as calcium and/or PTH levels^7,19,20^, and routine prophylactic supplementation in all patients undergoing total thyroidectomy regardless of symptoms or laboratory values^27–29^. The aim of this empirical approach is to prevent transient hypocalcaemia and facilitate early discharge, although it may result in overtreatment in a subset of patients. Several randomized clinical trials and previous meta-analyses^30–32^ have suggested that routine postoperative supplementation with calcium and vitamin D reduces the incidence of post-thyroidectomy hypocalcaemia. Ηowever, methodological limitations—including small sample sizes, co-interventions, and limited dose or subgroup stratification—have constrained the generalizability of these findings.

Since the publication of these analyses, additional randomized trials, including larger and more recent studies, have expanded the available evidence base, justifying a contemporary and methodologically rigorous re-evaluation. Moreover, evolving perioperative practices, such as supplementation protocols initiated before surgery and continued after surgery, raise the question of whether timing of supplementation influences clinical outcomes.

The objective of this network meta-analysis is to compare the effectiveness of perioperative calcium protocol supplementation, with or without vitamin D, in reducing postoperative biochemical and symptomatic hypocalcaemia and the need for intravenous calcium administration after total thyroidectomy. The effect of supplementation on length of hospital stay was evaluated as a secondary outcome.

## Materials and methods

This study was created in full adherence to updated PRISMA guidelines^33^ and AMSTAR 2 (assessing the methodological quality of systematic reviews) criteria for high-quality evidence synthesis^34^. The protocol was prospectively registered and is publicly available through the PROSPERO database (CRD420251068837).

### Data sources and literature search

An extensive literature search was conducted. The PubMed, Scopus, Cochrane Library, and Web of Science databases were searched by two independent authors without date constraints. Due to access restrictions, the Embase database was not included. Search strategies were developed using a series of keywords and medical subject headings terms in compliance with the PICO (Population, Intervention, Comparison, and Outcome) structure, including (thyroidectomy or thyroid surgery or post-thyroidectomy or post-thyroidectomy or after thyroidectomy) AND (dietary calcium or calcium or total calcium or gluconate calcium or carbonate calcium or vitamin D or calcitriol or alfacalcidol or caltrate or cholecalciferol or ergocalciferol) AND (hypocalcaemia or low calcium or hypoparathyroidism) AND (prevention or treatment or therapy or management). Additional relevant studies were identified by manually screening the reference lists of included articles and previous systematic reviews and meta-analyses. Study selection and data extraction were performed independently and in duplicate by two reviewers. Discrepancies were resolved through discussion. The last literature search was carried out on 7 May 2026. The full search strategies for each database are provided in the *supplementary methods*.

As this is a meta-analysis of previously published studies, ethics committee approval was not required. The review was limited to published articles that referred to adult patients (> 18 years old) and written in English.

### Eligibility criteria

Studies were eligible for inclusion if they met the following criteria: adult patients undergoing total or near-total or completion thyroidectomy for benign or malignant thyroid disease with/without neck dissection; postoperative or perioperative administration of oral calcium and/or vitamin D (cholecalciferol, alfacalcidol, or calcitriol) as a prophylactic intervention; and reporting on at least one of the following outcomes: symptomatic hypocalcaemia, defined as the occurrence of clinical symptoms such as perioral tingling, numbness of the extremities, tetany or positive Chvostek/Trousseau signs; biochemical hypocalcaemia, defined as postoperative abnormal serum calcium levels (most studies, for example, < 8.0 mg/dl); the need for intravenous calcium supplementation; and length of hospital stay. Exclusion criteria included studies with patients undergoing previous neck surgery, neck radiotherapy, parathyroid disease; those already taking calcium or vitamin D supplements before surgery; and those taking medications such as corticosteroids, diuretics, or bisphosphates that could influence serum calcium levels.

Studies were excluded if they were not randomized clinical trials. Study protocols without available outcome data or associated peer-reviewed publications were also excluded, as were studies not involving thyroidectomy-related calcium or vitamin D administration or addressing unrelated research questions. Trials evaluating exclusively preoperative supplementation were further excluded, as many of these studies investigated vitamin D monotherapy and demonstrated substantial heterogeneity in dosing regimens, formulations, and duration of administration. Finally, trials directly comparing preoperative with postoperative supplementation strategies were not included, as the aim of this analysis was to compare commonly used supplementation strategies rather than the timing of their administration.

### Outcomes of interest, definitions, and data extraction

For the purposes of the quantitative analysis, the predefined primary outcomes of interest were the incidence of clinical hypocalcaemia, as defined in each study, by the presence of symptoms following thyroidectomy; the incidence of biochemical hypocalcaemia, defined as depressed serum calcium levels below the laboratory threshold set in each study; and the need for intravenous calcium supplementation during the postoperative period, irrespective of the specific indication for its administration. Length of hospital stay was the secondary outcome. Baseline patient demographics, indication of surgery, intervention protocols, duration of therapy, type of surgery, and total length of follow-up were reported (*Table S1*).

For the purposes of this analysis, all interventions were categorized into three standardized prophylactic strategies. The first comprised oral calcium supplementation administered during the perioperative period, the second included oral calcium combined with vitamin D analogues (active analogues or native vitamin D), and the third strategy involved no routine prophylactic supplementation, with treatment initiated only in response to postoperative clinical or biochemical indications. These classifications formed the three treatment nodes evaluated in the network meta-analysis.

All data pertaining to the outcomes of interest were manually extracted from the included studies by two authors (V.G, D.P), with a third author (K.N) ensuring data accuracy and completeness, and entered into standardized Microsoft Excel® spreadsheets (Microsoft, Redmond, WΑ, USA) for further data tabulation.

### Risk of bias assessment

Assessment of the risk of bias in the included study data set was performed using the revised RoB 2 tool, developed by the Cochrane Collaboration^35^. This tool is specifically designed for the evaluation of the risk of bias in randomized clinical trials by judging the methodological quality of each study across five domains: adequacy of randomization process, the presence of deviations from intended interventions, the presence of missing data, bias due to incorrect measurement of outcomes, and selective reporting of results. For each of the components of the tool, a grade of either low, moderate (some concerns), or serious risk is allocated. The risk of bias assessment was performed independently by two authors (V.G, D.P), with a third author (K.N) refereeing any ensuing disputes.

### Certainty of evidence assessment

The certainty of evidence for each outcome and comparison was assessed using the GRADE (Grading of Recommendations Assessment, Development and Evaluation) approach adapted for network meta-analysis^36^. Certainty ratings considered risk of bias, inconsistency, indirectness, imprecision, and publication bias, as well as intransitivity and incoherence between direct and indirect evidence.

### Data synthesis and statistical analysis

Although a frequentist pairwise meta-analysis was specified at registration, a Bayesian network meta-analysis was undertaken to enable simultaneous comparison of prophylactic strategies and integration of direct and indirect evidence. The analysis was performed within a Bayesian framework, using the MetaInsight v6.4.0 software^37^, which implements Bayesian network meta-analysis (NMA) models through the gemtc and JAGS frameworks of R. Based on expected interstudy clinical and methodological heterogeneity, a random-effects model was preselected to estimate pooled odds ratios (ORs) and corresponding 95% credible intervals (cr.i.) for the three postoperative supplementation strategies: no supplementation, calcium alone, and calcium combined with vitamin D analogues. A Bayesian random-effects NMA was conducted using non-informative priors for treatment effects and heterogeneity, as implemented in the MetaInsight software (default priors). Markov Chain Monte Carlo (MCMC) simulation was performed with four chains, each run for 25 000 iterations following a burn-in of 5000. Convergence was assessed using trace plots and Gelman–Rubin shrink factors, with values below 1.05 considered indicative of adequate convergence.

Model fit was evaluated using the deviance information criterion (DIC) and the posterior mean residual deviance (dbar), with dbar values approximating the number of data points indicating good overall fit. Between-study heterogeneity was expressed as the between-study standard deviation. Consistency was examined by comparing the consistency (NMA) model with an unrelated mean effects (UME) model, with deviance comparison plots used to assess visually potential inconsistency between direct and indirect evidence. Local inconsistency was further explored through node-splitting analyses where applicable. Robustness and transitivity were assessed through predefined sensitivity analyses and evaluation of clinically relevant effect modifiers across the network.

Treatment rankings were summarized using the surface under the cumulative ranking curve (SUCRA), which quantifies the probability that each intervention is among the most effective options. SUCRA values range from 0 (least effective) to 1 (most effective), with higher values indicating a greater likelihood of ranking among the best-performing strategies. All pairwise treatment contrasts were presented in a league table showing pooled ORs and corresponding 95% cr.i. Statistical significance was inferred when the 95% confidence interval (c.i.) did not cross unity. Owing to the limited number of available studies and the lack of direct head-to-head comparisons across all treatment strategies, a network meta-analysis was not feasible for length of hospital stay; therefore, a pairwise random-effects meta-analysis was performed by calculating weighted mean difference using a random-effects model. A *P* value smaller than 0.05 was considered statistically significant.

To enhance clinical interpretability, approximate absolute risk reductions and numbers needed to treat were estimated using pooled baseline event rates from the no-supplementation groups and the corresponding ORs from the network meta-analysis. Additional sensitivity analyses were conducted to evaluate the robustness of the network estimates and explore potential sources of heterogeneity. These included exclusion of studies at high risk of bias (RoB 2), trials using native vitamin D preparations, and studies in which central neck dissection was routinely performed. These analyses were undertaken to assess the impact of methodological quality, pharmacologic heterogeneity, and surgical extent on the pooled treatment effects.

## Results

### Study selection and characteristics

**Fig. 1:**
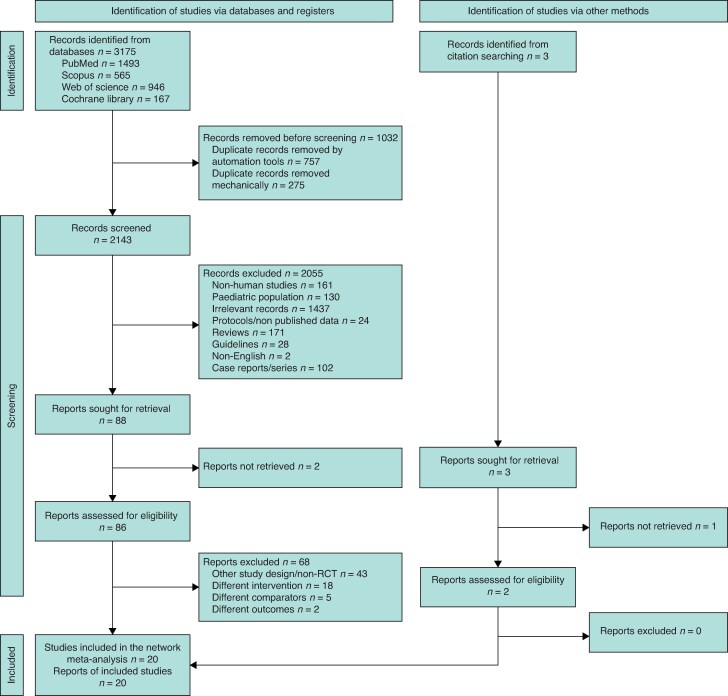
PRISMA 2020 flow diagram^33^ illustrating the study selection process, adapted from the PRISMA 2020 statement.^33^

The literature search identified 3178 records. After removal of duplicates and screening, 91 full-text articles were assessed for eligibility. Twenty randomized clinical trials^29,38–56^ met the inclusion criteria and were included in the quantitative synthesis. The study selection process is summarized in the PRISMA flow diagram (*Fig. 1*).

In total, 3669 patients were included in the network meta-analysis, and the baseline characteristics of the included studies are summarized in the *Table S1*. Mean patient age was broadly comparable across studies, with a predominance of women patients. The included studies evaluated the comparative effects of the three prophylactic approaches across the outcomes of interest. One three-arm trial^42^ reported zero events in two treatment arms for the outcome of postoperative intravenous calcium requirement. Because treatment effects cannot be estimated for contrasts with zero events in both arms within binomial network meta-analysis models, this study did not contribute to the outcome-specific network for intravenous calcium use. This affected only a single study and did not alter the network structure or pooled estimates.

### Assessment of methodological quality

Risk of bias was assessed using RoB 2 and is summarized in *Table 1*. Overall judgements ranged from low risk to high risk; concerns most frequently arose from limitations in the randomization process, insufficient reporting of allocation concealment, and from the selection of the reported results, because the lack of prospective registration in many studies precluded verification that all outcomes and analyses were prespecified. In line with RoB 2’s outcome-specific approach, the measurement of the outcome domain was judged separately when multiple primary outcomes were evaluated; in *Table 1* these are reported as biochemical hypocalcaemia/clinical hypocalcaemia/intravenous calcium requirement. Measurement bias was generally low for biochemical hypocalcaemia, whereas clinical hypocalcaemia and intravenous calcium use more often raised some concerns due to lack of blinding and symptom- or clinician-driven assessment.

**Table 1:** Risk of bias assessment of the included studies using the components of the RoB 2 tool^35^

Reference	Randomization process	Deviations from intended interventions	Missing outcome data	Outcome measurement	Selection of reported results	Overall
Langner *et al.*^38^	High risk	Some concerns	High risk	Low risk	Some concerns	High risk
Roh *et al*.^39^	Some concerns	Some concerns	Low risk	Low risk/low risk/low risk	Some concerns	Some concerns
Roh *et al*.^40^	Low risk	Low risk	Low risk	Low risk/low risk/low risk	Some concerns	Some concerns
Ravikumar *et al*.^41^	High risk	Low risk	Low risk	Low risk/low risk	Some concerns	High risk
Bellantone *et al.*^42^	Some concerns	Low risk	Low risk	Low risk/some concerns	Some concerns	Some concerns
Arer *et al*.^43^	Some concerns	Low risk	Low risk	Some concerns/some concerns	Some concerns	Some concerns
Choe *et al*.^44^	Some concerns	High risk	Low risk	Low risk/some concerns/high risk	Some concerns	High risk
Tartaglia *et al*.^45^	High risk	Low risk	Some concerns	Some concerns	Some concerns	High risk
Pisaniello *et al*.^46^	Some concerns	Some concerns	Some concerns	Low risk/low risk	Some concerns	Some concerns
Lee *et al*.^47^	Low risk	Low risk	Low risk	Low risk/low risk	Low risk	Low risk
Kurukahvecioglu *et al*.^29^	Some concerns	Some concerns	Low risk	Some concerns/some concerns	Some concerns	Some concerns
Li *et al*.^47^	Low risk	Low risk	Low risk	Low risk/low risk	Low risk	Low risk
Gkanis *et al*.^56^	Low risk	Low risk	Low risk	Low risk/low risk/low risk	Low risk	Low risk
Mercante *et al*.^49^	Some concerns	Low risk	Low risk	Low risk/low risk/low risk	Some concerns	Some concerns
Hao *et al*.^50^	Some concerns	Low risk	Low risk	Low risk/low risk	Low risk	Some concerns
Jaan *et al*.^51^	High risk	Low risk	Low risk	Low risk/low risk/low risk	Some concerns	High risk
Nemade *et al.*^52^	High risk	Low risk	Low risk	Low risk/some concerns/some concerns	Some concerns	High risk
Ghafouri *et al*.^53^	Low risk	Low risk	Low risk	Some concerns/some concerns	Some concerns	Some concerns
El-Shinawi *et al*.^54^	Some concerns	Low risk	Low risk	Low risk/some concerns/some concerns	Some concerns	Some concerns
Li *et al*.^55^	Low risk	Some concerns	Low risk	Low risk/some concerns/Some concerns	Low risk	Some concerns

For studies reporting multiple primary outcomes, the measurement of the outcome domain is presented in the order biochemical hypocalcaemia/clinical hypocalcaemia/intravenous calcium requirement.

### Certainty of evidence (GRADE)

The certainty of evidence was assessed at the level of network estimates, which were considered the primary estimates of effect in the absence of important incoherence. Risk of bias assessments were based on the RoB 2 tool. Inconsistency and incoherence were evaluated using heterogeneity measures, node-splitting analyses, and comparison of consistency and inconsistency models. Indirectness and intransitivity were assessed by examining the similarity of study populations, interventions, comparators, and outcomes across the network. Imprecision was evaluated based on the width of the 95% cr.i. and their relation to the line of no effect, taking into account clinical relevance. Publication bias was assessed qualitatively. Overall, the certainty of evidence ranged from moderate to low across comparisons. A summary of the certainty of evidence is presented in *Table 2*.

**Table 2:** GRADE assessment of certainty of evidence for network meta-analysis estimates across outcomes and comparisons

Outcome	Comparison	Network estimate OR	Risk of bias	Inconsistency	Indirectness/intransitivity	Incoherence	Imprecision	Publication bias	Final Certainty
Clinical hypocalcaemia	Ca + VitD *versus* no treatment	0.31 (0.17, 0.51)	Serious	Not serious	Not serious	Not serious	Not serious	Undetected	Moderate
	Ca *versus* no treatment	0.60 (0.26, 1.26)	Serious	Not serious	Not serious	Not serious	Serious	Undetected	Low
	Ca + VitD *versus* Ca	0.52 (0.25, 1.04)	Serious	Not serious	Not serious	Not serious	Serious	Undetected	Low
Biochemical hypocalcaemia	Ca + VitD *versus* no treatment	0.27 (0.17, 0.42)	Serious	Not serious	Not serious	Not serious	Not serious	Undetected	Moderate
	Ca *versus* no treatment	0.62 (0.33, 1.19)	Serious	Not serious	Not serious	Not serious	Serious	Undetected	Low
	Ca + VitD *versus* Ca	0.44 (0.22, 0.83)	Serious	Not serious	Not serious	Not serious	Not serious	Undetected	Moderate
i.v. calcium supplementation	Ca + VitD *versus* no treatment	0.15 (0.05, 0.32)	Serious	Not serious	Not serious	Not serious	Not serious	Undetected	Moderate
	Ca *versus* no treatment	0.43 (0.12, 1.17)	Serious	Not serious	Not serious	Not serious	Serious	Undetected	Low
	Ca + VitD *versus* Ca	0.36 (0.10, 1.00)	Serious	Not serious	Not serious	Not serious	Serious	Undetected	Low

Values in parentheses are 95% credible intervals. Certainty of evidence was assessed using the GRADE approach adapted for network meta-analysis. Because all included studies were randomized clinical trials, certainty started at high and was rated down according to risk of bias, inconsistency, indirectness/intransitivity, incoherence, imprecision, and publication bias. Network estimates were considered the best estimates of effect because no important incoherence between direct and indirect evidence was identified. GRADE, Grading of Recommendations Assessment, Development and Evaluation; OR, odds ratio; Ca, calcium; VitD, vitamin D; i.v., intravenous.

### Clinical hypocalcaemia

**Fig. 2:**
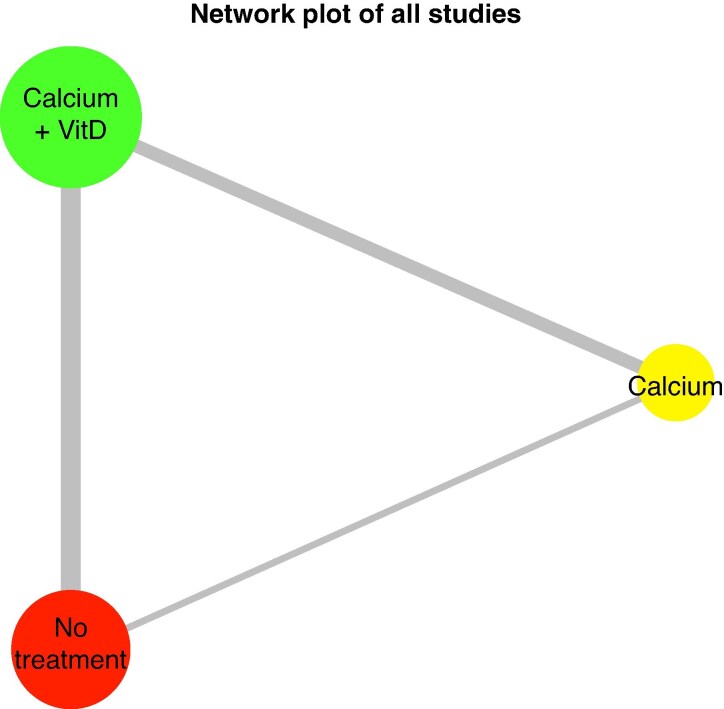
Triangle-shaped network plot with each node representing one of the involved treatment strategies The size of the node and the thickness of the edges represent the number of studies examining a specific treatment pair. Network plots for all three outcomes were visually identical.

A total of 18 randomized clinical trials^29,39,40,42–56^ incorporating 3424 patients contributed data for this analysis. Among them, 15 studies were two-armed, and 3 studies were multiarmed, forming a well-connected three-node triangular network involving no supplementation, calcium alone, and calcium with vitamin D analogues (*Fig. 2*). Across the included trials, the pooled event rates for clinical hypocalcaemia were 22.8% in the no-supplementation groups, 24.5% in the calcium-only groups, and 13.1% in the calcium plus vitamin D groups, corresponding to an estimated absolute risk reduction of approximately 14.4% and a number needed to treat (NNT) of approximately seven for combination therapy *versus* no supplementation.

**Fig. 3:**
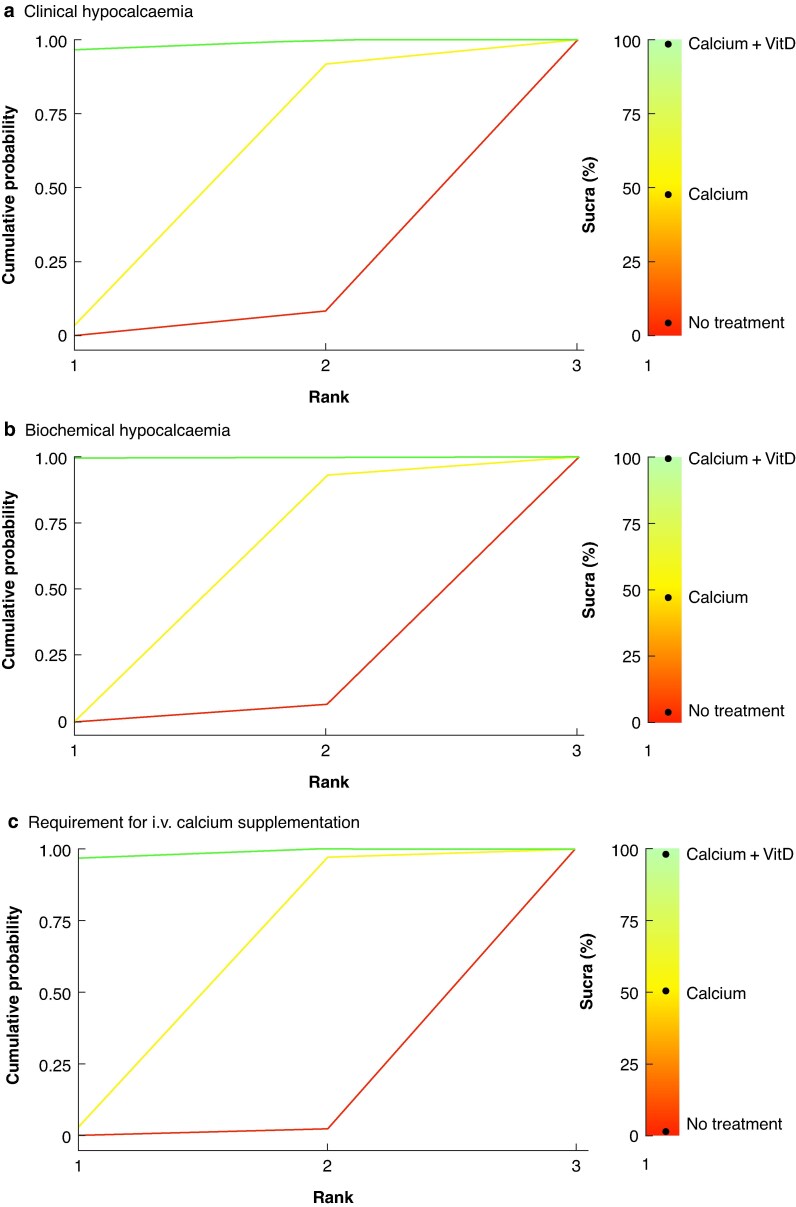
Ranking results for the development of clinical hypocalcaemia, biochemical hypocalcaemia, and requirement for intravenous calcium supplementation **a** clinical hypocalcaemia**, b** biochemical hypocalcaemia**, c** requirement for i.v. calcium supplementation, using the Litmus Rank-O-Gram, demonstrating the cumulative probability for each treatment to be ranked as first, second, or third, along with relevant SUCRA values. Higher cumulative probabilities and SUCRA values indicate a greater likelihood that an intervention ranks among the most effective strategies. i.v., intravenous.

In the Bayesian random-effects network meta-analysis, combination therapy with calcium plus vitamin D analogues was associated with a substantially lower risk of clinical hypocalcaemia compared with no supplementation (OR 0.31, 95% cr.i. 0.17 to 0.51). Combination therapy also demonstrated superiority over calcium alone (OR 0.52, 95% cr.i. 0.25 to 1.04), although the credible interval marginally crossed unity. Calcium monotherapy did not significantly reduce clinical hypocalcaemia rates relative to no supplementation (OR 0.60, 95% cr.i. 0.26 to 1.26) (*Table 3*). Treatment ranking based on cumulative ranking probabilities (*Fig. 3a*) indicated that combination therapy had the highest probability of being the most effective strategy for preventing clinical hypocalcaemia, as reflected by the most favourable ranking distribution and the highest SUCRA value, followed by calcium alone, whereas no supplementation consistently ranked last.

**Table 3:** League table showing pooled odds ratios (95% credible intervals) for each pairwise comparison between the evaluated supplementation strategies across the assessed outcomes

	Calcium	Calcium + VitD	No treatment
**Clinical hypocalcaemia**			
Calcium		0.52 (0.25, 1.04)	1.67 (0.79, 3.77)
Calcium + VitD	1.93 (0.96, 4.00)		3.22 (1.96, 5.87)
No treatment	0.60 (0.26, 1.26)	0.31 (0.17, 0.51)	
**Biochemical hypocalcaemia**			
Calcium		0.44 (0.22, 0.83)	1.61 (0.84, 3.04)
Calcium + VitD	2.28 (1.21, 4.53)		3.66 (2.40, 5.92)
No treatment	0.62 (0.33, 1.19)	0.27 (0.17, 0.42)	
**Requirement of intravenous calcium supplementation**			
Calcium		0.36 (0.10, 1.00)	2.32 (0.85, 8.33)
Calcium + VitD	2.79 (1.00, 9.64)		6.5 (3.13, 21.77)
No treatment	0.43 (0.12, 1.17)	0.15 (0.05, 0.32)	

Values are odds ratios (95% credible intervals). VitD, vitamin D.

### Biochemical hypocalcaemia

Twelve studies^38–41,44,48,49,51,52,54–56^ totalling 2043 patients reported data on abnormally depressed calcium levels in the postoperative period. Nine of these studies were two-armed and three were multiarmed, with a well-connected triangle-shaped network. Across the included trials, the pooled event rates for biochemical hypocalcaemia were 37.2% in the no-supplementation groups, 36.6% in the calcium-only groups, and 15.8% in the calcium plus vitamin D groups, corresponding to an estimated absolute risk reduction of approximately 23.4% and an NNT of approximately four for combination therapy *versus* no supplementation. Combination therapy substantially reduced the odds of postoperative biochemical hypocalcaemia compared with no supplementation (OR 0.27, 95% cr.i. 0.17 to 0.42) and calcium alone (OR 0.44, 95% cr.i. 0.22 to 0.83). Calcium monotherapy demonstrated a non-significant trend towards benefit compared with no supplementation (OR 0.62, 95% cr.i. 0.33 to 1.19) (*Table 3*). Treatment ranking based on cumulative probability plots (*Fig. 3b*) showed that combination therapy had the highest probability of being the most effective strategy, followed by calcium alone, whereas no supplementation consistently ranked lowest.

### Requirement for intravenous calcium supplementation

Seventeen studies^29,39–44,46,47,49–56^ involving 3012 patients contributed data to the evaluation of postoperative intravenous calcium requirements. Following exclusion of a study with incomplete arm-level data^47^ and a study with non-estimable treatment effects^42^, the resulting three-node network remained fully connected. Across the included trials, the pooled event rates for postoperative intravenous calcium use were 11.6% in the no-supplementation groups, 16.0% in the calcium-only groups, and 4.0% in the calcium plus vitamin D groups, corresponding to an estimated absolute risk reduction of approximately 9.7% and an NNT of approximately ten for combination therapy *versus* no supplementation. Combination therapy demonstrated the greatest protective effect, significantly reducing the odds of intravenous calcium use compared with no supplementation (OR 0.15, 95% cr.i. 0.05 to 0.32) and outperforming calcium alone (OR 0.36, 95% cr.i. 0.10 to 1.00). Calcium monotherapy showed a non-significant trend towards benefit *versus* no supplementation (OR 0.43, 95% cr.i. 0.12 to 1.17) (*Table 3*). Ranking analyses consistently placed combination therapy as the most effective strategy, followed by calcium alone, with no supplementation ranking lowest (*Fig. 3c*).

### Length of hospital stay

**Fig. 4:**
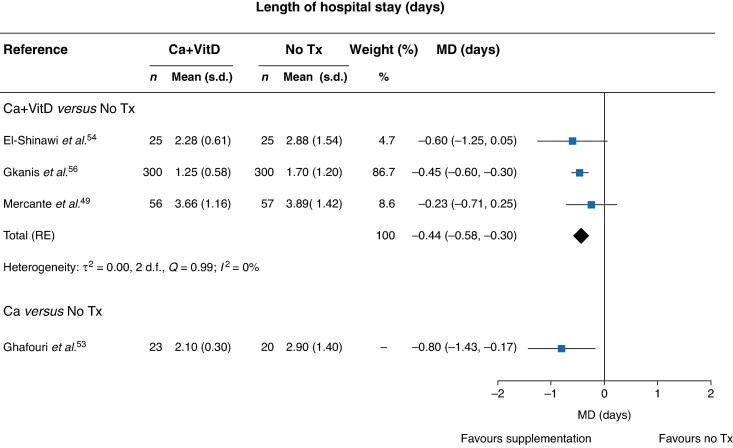
Length of hospital stay (days) Forest plot of the pairwise random-effects meta-analysis comparing calcium plus vitamin D analogue supplementation *versus* no routine supplementation. Three randomized trials were pooled to estimate the mean difference in length of hospital stay, whereas one additional trial comparing calcium alone *versus* no supplementation is presented separately as a single-study estimate. Negative values favour supplementation. Error bars represent 95% confidence intervals. Ca, calcium; VitD, vitamin D; Tx, treatment; MD, mean difference; s.d., standard deviation; d.f., degrees of freedom.

Four randomized clinical trials^49,53,54,56^ reported numerical data on length of hospital stay. Three trials comparing calcium plus vitamin D analogue supplementation with no routine supplementation were pooled in a random-effects pairwise meta-analysis, demonstrating a significantly shorter length of hospital stay with combination therapy (mean difference (MD) 0.44 days, 95% c.i. −0.58 to −0.30), with no observed between-study heterogeneity (*I*^2^ = 0%), acknowledging the limited number of contributing studies. One additional trial comparing calcium monotherapy with no supplementation also reported a shorter length of hospital stay (MD −0.80 days) but was not pooled due to the single-study design (*Fig. 4*). A further randomized trial^29^ reported a statistically significant reduction in length of hospital stay (*P* < 0.001) without providing numerical data and was therefore included only in the qualitative synthesis.

### Consistency and transitivity analysis

Local inconsistency was assessed using node-splitting models for all three primary outcomes. No statistically significant disagreement between direct and indirect evidence was detected across the networks. For clinical hypocalcaemia, none of the treatment contrasts showed significant inconsistency (all *P* > 0.050), although borderline differences were observed for calcium *versus* calcium plus vitamin D (*P* = 0.065) and calcium *versus* no treatment (*P* = 0.099), without influencing the pooled network estimates. Similarly, no evidence of inconsistency was identified for biochemical hypocalcaemia (*P* 0.058–0.111) or intravenous calcium supplementation (*P* 0.240–0.930), with direct and indirect estimates remaining directionally concordant and fully aligned with the network summary effects.

Robustness was evaluated through a predefined sensitivity analysis excluding trials with perioperative supplementation^51–53,55^. Following this restriction, all outcome-specific networks remained connected, allowing node-splitting for a single comparison per outcome. Borderline inconsistency was observed for calcium *versus* no supplementation in clinical hypocalcaemia (*P* = 0.040) and for calcium *versus* calcium plus vitamin D in biochemical hypocalcaemia (*P* = 0.037), whereas no inconsistency was detected for intravenous calcium use (*P* = 0.840). Importantly, these findings did not materially affect pooled network estimates or treatment rankings, with combination therapy consistently remaining the most effective strategy across all outcomes, supporting the robustness of the primary conclusions.

Moreover, a sensitivity analysis excluding trials judged as overall high risk of bias according to the RoB 2 assessment^38,41,44,45,51,52^, as well as trials using native vitamin D preparations^29,43,46,47,52^, was performed to evaluate the robustness of the network estimates. Following these exclusions, pooled estimates remained stable across all outcomes, with no meaningful changes in the direction of treatment effects or intervention ranking. Credible intervals widened slightly in some comparisons owing to the reduced number of studies, but the overall interpretation of the results was not materially altered. Detailed results are presented in *Tables S2*, *S3*.

Transitivity was assessed by examining clinically relevant effect modifiers across treatment nodes. Age and hyperthyroidism as an indication for surgery were similarly distributed (analysis of variance, *P* = 0.810; χ^2^, *P* = 0.078), supporting baseline comparability. In contrast, malignancy and lymph node dissection exhibited significant between-node imbalances (χ^2^, both *P* < 0.001), with the calcium-only group including a higher proportion of malignant cases and more frequent lymph node dissection, reflecting greater surgical complexity and baseline hypocalcaemia risk. However, all included studies were randomized clinical trials, and there was no indication that malignancy status or surgical extent influenced allocation to supplementation strategy within individual trials. A sensitivity analysis excluding studies with routine or predominant central neck dissection^39,44,47,48,50,55^ yielded comparable estimates, with no meaningful changes in effect direction or treatment ranking. Combination therapy remained the most effective strategy indicating that internal validity and interpretability of the network were not materially compromised.

### Measures of fit and model convergence

Across all three primary outcomes, the Bayesian random-effects network meta-analysis demonstrated good model fit, with close agreement between fitted and observed values. DIC values were 72.1 for clinical hypocalcaemia, 48.8 for biochemical hypocalcaemia, and 67.6 for intravenous calcium requirement, with corresponding dbar of 40.0 (39 data points), 27.7 (27 data points), and 41.5 (36 data points). Between-study heterogeneity was moderate for clinical hypocalcaemia (τ = 0.81, 95% cr.i. 0.41 to 1.35), lower for biochemical hypocalcaemia (τ = 0.50, 95% cr.i. 0.10 to 1.03), and higher for intravenous calcium use (τ = 1.06, 95% cr.i. 0.27 to 2.37), remaining within acceptable bounds for random-effects modelling.

Comparison of residual deviances between the consistency (NMA) and inconsistency (UME) models showed that data points across all outcomes were aligned closely with the line of equality, indicating good agreement and no meaningful inconsistency in the network. Per-arm residual deviances close to 1 and absence of high-leverage observations indicate that no individual study exerted disproportionate influence on the pooled estimates (*Fig. S1*).

Convergence was excellent for all outcomes: Gelman–Rubin shrink factors approached 1.00 for every parameter after approximately 10 000 iterations, trace plots demonstrated adequate chain mixing across all four chains, and posterior densities were unimodal and symmetric (*Fig. S2*). Collectively, these diagnostics confirm reliable MCMC performance and robust estimation of relative treatment effects across the network.

## Discussion

Postoperative hypocalcaemia remains the most frequent and clinically significant complication following total thyroidectomy, contributing to a prolonged length of hospital stay, increased healthcare utilization, and substantial patient discomfort^57–59^. Although early postoperative serum calcium and PTH measurements are widely used to guide selective supplementation^21,60–63^, these biomarkers show variable sensitivity and are not universally available, limiting their reliability as sole predictors of hypocalcaemia^19,64–66^. Conversely, routine perioperative supplementation with calcium, with or without vitamin D, has been proposed to mitigate transient hypocalcaemia and support early discharge pathways^67–70^, yet its adoption in clinical practice remains heterogeneous owing to concerns regarding overtreatment and uncertainty about its comparative effectiveness.

By integrating direct and indirect evidence, this network meta-analysis demonstrated that combination therapy consistently ranked as the most effective approach across all clinically relevant outcomes. In contrast, calcium monotherapy showed only a modest, non-significant trend towards benefit, whereas no routine supplementation was uniformly the least effective strategy. The consistency of treatment effects across sensitivity and diagnostic analyses supports the robustness of these findings. Whereas the results support the use of routine combination therapy in early postoperative care, further studies evaluating long-term outcomes such as permanent hypoparathyroidism are warranted.

A modest but statistically significant reduction in length of hospital stay was also observed with supplementation, although this finding is based on limited data. In the context of contemporary endocrine surgical practice and high-volume centres where same-day or short-stay thyroidectomy pathways are increasingly implemented, routine supplementation may still facilitate earlier discharge by reducing early postoperative hypocalcaemia symptoms, limiting the need for intensive biochemical monitoring—including outpatient settings—and decreasing the likelihood of readmission or intravenous calcium administration^30,32^. Consequently, standardized supplementation protocols may also contribute to reductions in healthcare resource utilization and overall treatment costs^49,71^.

The superiority of combined calcium and vitamin D supplementation is biologically plausible, as postoperative reductions in PTH impair both renal and intestinal calcium handling^72^. By enhancing active intestinal calcium absorption, vitamin D may compensate for reduced parathyroid reserve, whereas calcium monotherapy, which relies largely on passive absorption, may be insufficient in the early postoperative period^73–75^

Several meta-analyses have examined prophylactic calcium and vitamin D supplementation after thyroidectomy, despite substantial heterogeneity in dosing, timing, and treatment algorithms. Sanabria *et al*.^31^, in a meta-analysis of 15 randomized clinical trials (3037 patients), reported that calcium plus vitamin D significantly reduced symptomatic (risk difference (RD) −0.25, 95% c.i. −0.32 to −0.18) and biochemical hypocalcaemia (RD −0.24, 95% c.i. −0.31 to −0.17), as well as intravenous calcium requirements, without reducing permanent hypoparathyroidism or showing additional benefit from higher calcium doses. Similarly, Xing *et al*.^32^, analysing ten randomized trials (1620 patients), found that calcium supplementation reduced postoperative hypocalcaemia (OR 0.48, 95% c.i. 0.31 to 0.74), with a greater effect when combined with vitamin D (OR 0.21, 95% c.i. 0.11 to 0.40), and further decreased intravenous calcium use (OR 0.26, 95% c.i. 0.10 to 0.69) Against this background, the present network meta-analysis directly comparing the three most commonly used prophylactic strategies within a unified framework identified only a non-significant trend towards benefit for calcium monotherapy compared with no supplementation, indicating a more modest and less consistent effect than previously reported.

A recent network meta-analysis by Ren *et al*.^76^, including 27 randomized trials and 3382 patients, compared a broad range of prophylactic strategies after total thyroidectomy. Using a frequentist random-effects framework, the authors reported the greatest reduction in symptomatic hypocalcaemia with teriparatide (relative risk (RR) 0.18, 95% c.i. 0.03 to 0.98), whereas combination therapy with calcium plus vitamin D3 or activated vitamin D3 also significantly reduced symptomatic and biochemical hypocalcaemia compared with placebo. Although this analysis provides valuable comparative insights, several less commonly used interventions were supported by limited trial data, restricting the strength of certain comparisons. In contrast, the present network meta-analysis focuses on the three most widely adopted strategies in routine practice, enabling a more consistent and clinically pragmatic comparison. Differences in scope, analytical framework, and inclusion criteria should be considered when interpreting these findings.

Although combination therapy demonstrated clear clinical benefit, safety considerations remain relevant when implementing routine supplementation protocols. Short-term perioperative calcium and vitamin D supplementation is generally well tolerated, with the risk of nephrolithiasis from brief courses considered negligible^77^. Nonetheless, calcium supplementation may increase urinary calcium excretion, and vitamin D may exacerbate stone risk in individuals predisposed to hypercalciuria^72,78^. Rare cases of clinically significant hypercalcemia following high-dose supplementation have also been reported, underscoring the importance of biochemical monitoring^79^. These findings have clear implications for postoperative care after total thyroidectomy. Beyond confirming existing practices, this analysis provides a quantitative and clinically focused comparison of supplementation strategies, supporting a more standardized, protocol-based approach to care. The consistent superiority of combined calcium and vitamin D supplementation suggests that routine use of this regimen can reduce early hypocalcaemia, limit intravenous calcium requirements, and facilitate safe implementation of outpatient or short-stay pathways. Importantly, these results may help reduce the substantial variation currently observed in postoperative supplementation practices. Routine supplementation may also support risk-adapted strategies, particularly in patients undergoing more extensive surgery, while offering a pragmatic alternative to PTH-guided stratification in settings where timely measurement is unavailable. This approach could further reduce intensive biochemical monitoring, readmissions related to transient hypocalcaemia, and overall healthcare costs.

This study has several notable strengths. It provides a focused network meta-analysis of randomized trials evaluating widely used prophylactic strategies in routine practice, incorporating recent high-quality trials. The Bayesian random-effects framework enabled integration of direct and indirect evidence, with consistent findings across model diagnostics and sensitivity analyses. The well-connected triangular network supported robust comparative estimates, whereas exclusion of trials with exclusively preoperative supplementation reduced clinical heterogeneity and improved comparability between strategies. Compared with previous analyses including broader or less consistent interventions, this study provides more pragmatic and clinically applicable estimates. Together, these methodological features strengthen the interpretability of the results and enhance their relevance to everyday clinical decision-making.

Several limitations warrant consideration. Although only randomized clinical trials were included, residual heterogeneity persisted across studies in patient characteristics, surgical indications, and supplementation protocols, including dosing, formulation, and duration of calcium and vitamin D administration. Pharmacologic heterogeneity within the vitamin D node also remained, as both active and native preparations were included despite differences in pharmacokinetics and onset of action. In addition, variation in surgical extent may have contributed to clinical heterogeneity across studies. However, sensitivity analyses excluding trials using native vitamin D and studies with routine or predominant central neck dissection yielded consistent findings, supporting the robustness of the overall conclusions. Definitions of biochemical hypocalcaemia and thresholds for intravenous calcium use were not fully uniform, potentially introducing variability in outcome assessment. Owing to limited study numbers and sparse direct comparisons, a network meta-analysis for length of hospital stay was not feasible, and estimates for this secondary outcome—derived from a small subset of trials—should be interpreted with caution. In addition, the analysis focused on early postoperative outcomes and did not evaluate long-term endpoints such as permanent hypoparathyroidism, which represents the most clinically significant complication after thyroidectomy, as well as late adverse events. Finally, exclusion of trials using vitamin D monotherapy or exclusively preoperative supplementation may limit generalizability to settings where alternative prophylactic strategies are employed.

## Supplementary Material

zrag093_Supplementary_Data

## Data Availability

All data analysed in this study were derived from previously published studies. No new data were generated. Extracted data are available from the corresponding author upon reasonable request.
