# Supplementary material for: Effectiveness of perioperative calcium and vitamin D supplementation in preventing post-thyroidectomy hypocalcaemia: network meta-analysis of randomized trials
Source: BJS Open. 2026 Jul 3;10(4):zrag093. doi: 10.1093/bjsopen/zrag093 (PMC13330921; doi:10.1093/bjsopen/zrag093)
Supplement: zrag093_Supplementary_Data [file zrag093_supplementary_data.zip › Supplementary_Material.docx]

**Title: Effectiveness of Perioperative Calcium and Vitamin D Supplementation in Preventing Post-Thyroidectomy Hypocalcemia: Network Meta-Analysis of Randomized Trials.**

**Authors:** Vasileios Gkanis^1^, Dimitrios Papaconstantinou^2^, Sophocles Lanitis^1^, Nikolaos Dafnios^3^, Ioannis Papakonstantinou^3^, Konstantinos Nastos^2^

**Affiliations**

1. Second Surgical Department and Unit of Surgical Oncology “Korgialeneio – Benakeio”, Hellenic Red Cross, General Hospital of Athens,

Athanasaki 1 and Erythrou Stavrou, Athens, Greece 11526

2. Third Department of Surgery, National and Kapodistrian University of Athens (NKUA), Attikon University Hospital, Rimini 1, Athens, Greece 12462

3. Second Department of Surgery, National and Kapodistrian University of Athens (NKUA), Aretaieio Hospital, Vasilissis Sofias 76, Athens, Greece 11528

**Corresponding author:** Vasileios Gkanis MD MSc

2nd Surgical Department and Unit of Surgical Oncology,

“Korgialeneio – Benakeio” Hellenic Red Cross, General Hospital of Athens,

Athanasaki 1 and Erythrou Stavrou, Athens, Greece 11526

E-mail: vasilisganis021@gmail.com, Work phone number: +30 2132068735

ORCID ID: 0000-0002-4103-216X

**Supplementary Materials - Index**

**Supplementary Methods**

- Full electronic search strategies for PubMed, Scopus, Web of Science, Cochrane Library Page: 2-3

**Supplementary Tables**

- Supplementary Table S1: Baseline characteristics of included studies.

Page: 4-7

- Supplementary Table S2: Sensitivity analysis excluding trials judged as overall high risk of bias according to the RoB 2 assessment. Page: 9
- Supplementary Table S3: Sensitivity analysis excluding studies using native Vitamin D preparations. Page:10
- Supplementary Table S4. Raw study-level data used for the network meta-analysis. Page: 11

**Supplementary Figures**

- Supplementary Figure S1: Model diagnostics: deviance and residual plots

Page: 12

- Supplementary Figure S2: Convergence diagnostics: Gelman–Rubin shrink factor plots, trace plots, posterior density plots Page: 13

**Supplementary Methods**

# **Final PICO-Based Search Strategies for Systematic Review**

The following search strategies are structured according to the PICO framework and are intended for use in a systematic review/meta-analysis regarding the effect of calcium and vitamin D administration after thyroidectomy on postoperative hypocalcemia. These strategies include both keywords and MeSH terms (for PubMed).

## **PubMed (with MeSH + Keywords)**

(Population/Setting)
("Thyroidectomy"[MeSH Terms] OR thyroidectomy OR "thyroid surgery" OR postthyroidectomy OR "post-thyroidectomy" OR "after thyroidectomy")
AND
(Intervention)
("Calcium, Dietary"[MeSH Terms] OR "Calcium"[MeSH Terms] OR dietary calcium OR calcium OR "total calcium" OR "gluconate calcium" OR "carbonate calcium" OR
"Vitamin D"[MeSH Terms] OR vitamin D OR calcitriol OR alfacalcidol OR caltrate OR cholecalciferol OR ergocalciferol)
AND
(Condition/Outcome)
("Hypocalcemia"[MeSH Terms] OR hypocalcemia OR "low calcium" OR
"Hypoparathyroidism"[MeSH Terms] OR hypoparathyroidism)
AND
(Approach/Purpose)
(prevention OR treatment OR therapy OR management)

## **Scopus**

TITLE-ABS-KEY("thyroidectomy" OR "thyroid surgery" OR postthyroidectomy OR "post-thyroidectomy" OR "after thyroidectomy")
AND
TITLE-ABS-KEY(dietary calcium OR calcium OR "total calcium" OR "gluconate calcium" OR "carbonate calcium" OR "vitamin D" OR calcitriol OR alfacalcidol OR caltrate OR cholecalciferol OR ergocalciferol)
AND
TITLE-ABS-KEY(hypocalcemia OR "low calcium" OR hypoparathyroidism)
AND
TITLE-ABS-KEY(prevention OR treatment OR therapy OR management)

## **Web of Science**

TS=("thyroidectomy" OR "thyroid surgery" OR postthyroidectomy OR "post-thyroidectomy" OR "after thyroidectomy")
AND
TS=(dietary calcium OR calcium OR "total calcium" OR "gluconate calcium" OR "carbonate calcium" OR "vitamin D" OR calcitriol OR alfacalcidol OR caltrate OR cholecalciferol OR ergocalciferol)
AND
TS=(hypocalcemia OR "low calcium" OR hypoparathyroidism)
AND
TS=(prevention OR treatment OR therapy OR management)

## **Cochrane Library**

("thyroidectomy" OR "thyroid surgery" OR postthyroidectomy OR "post-thyroidectomy" OR "after thyroidectomy")
AND
(dietary calcium OR calcium OR "total calcium" OR "gluconate calcium" OR "carbonate calcium" OR "vitamin D" OR calcitriol OR alfacalcidol OR caltrate OR cholecalciferol OR ergocalciferol)
AND
(hypocalcemia OR "low calcium" OR hypoparathyroidism)
AND
(prevention OR treatment OR therapy OR management)

**Supplementary Tables**

**Supplementary Table S1:** Study characteristics, interventions, and reported outcomes of randomized trials included in the network meta-analysis.

| **Study (year, country)** | **Number of patients (n)** | **Age (years, mean ± SD)** | **Female/Male n (%)** | **Malignancy n (%)** | **Hyperthyroidism n (%)** | **Surgical Procedure n, (%)** | **Supplementation strategies n, (%)** | **Duration of therapy** | **Definition of hypocalcemia** | **Outcomes / Comments** | **Follow-up (months)** |
| --- | --- | --- | --- | --- | --- | --- | --- | --- | --- | --- | --- |
| Langner et al, 2016, Brazil | 47 | 52.1 **±** 12.8 | 44/3 (93.6%/6.4%) | 23 (48.9%) | 6 (12.8%) | 47TT (100%) | Intervention: Ca 3gr/day, Controls: No treatment | 6 days postoperatively | <1.1mmol/L | Less biochemical hypocalcemia at POD1 when Ca administration (p=0.025), significant grater rate of symptomatic hypocalcemia in patients who did not receive calcium at POD1, POD7, POD90. | 6 months |
| Roh et al, 2006, Korea | 90 | 47 **±** 2.23 | 73/17 (81.1%/18.9%) | 75 (83.3%) | 5 (5.6%) | 71TT (78.8%) /19MRND (21.2%) | Intervention: Ca 3gr/day + VitD 1gr/day, Controls: No treatment | 14 days postoperatively | <8mg/dl | Lower incidence of hypocalcemia in the supplementation group (P<0.02), lower need for iv Ca (p=0.02), total Ca values significantly lower in the no supplement group at POD2, POD3, POD7. | 6 months |
| Roh et al, 2009, Korea | 148 | 48.5 **±** 12.3 | 120/28 (81.1%/18.9%) | 148 (100%) | 0 | 112TT + CND (75.7%)/ 36 TT+CND+MRND (24.3%) | GroupA: 3 gr Ca+ 1μg Alphacalcidol, Group B: 3 gr Ca, Group C: No treatment | 14 days postoperatively | <8mg/dl | Lowest incidence of symptomatic and laboratory hypocalcemia in Group A (p=0.002, p<0.001), more severe symptoms in Group C, iv Ca in 7 pts of Group C vs 0 in Group A, statistical higher Ca values at POD1, POD2, POD3, POD7 in pts with supplementation. | 36 months (24-46) |
| Ravikumar et al, 2017, India | 208 | 38.93 (18-65)* | 178/30 (85.6%/14.4%) | 0 | 0 | 208 TT (100%) | GroupA: No treatment, GroupB: Ca 2gr/day, GroupC: Ca 2gr/day + 1mcg Calcitriol/day, GroupD: Ca 2gr/day + 1mcg Calcitriol/day + Cholecalciferol 60000IU/day | 5 days postoperatively | <8mg/dl | During the first three postoperative days, Groups A and B had lower serum calcium levels than Groups C and D (p < 0.05), with no difference between Groups C and D (p >0.05). The calcium values on POD4-5 were comparable between all groups (p > 0.05). More iv Ca required in Group A and B (p=0.001). | 6 months |
| Bellantone et al, 2002, Italy | 79 | 49 **±** 13.3 | 61/18 (77.2%/22.8%) | 18 (22.8%) | 8 (10.1%) | 79TT (100%) | GroupA: No treatment, GroupB: Ca 3gr/day, GroupC: Ca 3gr/day + 1μg Calcitriol | 7 days postoperatively (except 8 pts) | <8mg/dl | Routine supplementation therapy with Ca or Vitamin D effectively prevents symptomatic hypocalcemia after total thyroidectomy and may allow for a safe early discharge. | 6 months |
| Arer et al, 2016, Turkey | 106 | 45.29 **±** 11.96 | 88/18 (83.0%/17.0%) | 16 (15.1%) | 22 (20.8%) | 100TT (94.3%)/ 6TT+ Neck Dissection (5.6%) | Intervention: pts<70kg: 5gr Ca carbonate+1760 IU VitD3/day, pts>70kg: 7.5gr Ca carbonate + 2640IU/day, Controls: No treatment | POD day 1-7 + 7 days decreased doses | < 8.5mg/dl | Prophylactic therapy can prevent postoperative hypocalcemia, is cost-effective and safe. 12h PTH and 24h PTH can predict hypocalcemia. | 2-16 months |
| Choe et al, 2010, Korea | 306 | 48.6 **±** 9.4 | 230/76 (75.2%/24.8%) | 306 (100%) | 0 | 306TT+ CND (100%) | GroupA1: Ca 3gr/d + Cholecalciferol 20μg/day, GroupA2: Ca 3gr/d + Calcitriol 0.5μg/day, GroupB1: like A1 on demand, GroupB2: like A2 on demand | Postoperatively (most pts for 2 weeks) | < 8mg/dl | Less laboratory and symptomatic hypocalcemia in patients with routine supplementation regardless of vitamin D type. No difference in number of pts with severe symptoms who needed iv Ca among group A and B. | NA |
| Tartaglia et al, 2005, Italy | 417 | 51 (38-61)* | 346/71 (83.0%/17.0%) | 86 (20.6%) | 55 (13.2%) | 417TT (100%) | GroupA: 1.5gr Ca + 1μg Calcitriol/day, GroupB: 1.5gr Ca + 2μg Calcitriol/day, GroupC: 1.5gr Ca/day | 15 days postoperatively with gradual reduction | NA | 2 μg of calcitriol / day after thyroidectomy significantly reduces the risk of severe hypocalcemia. | 6 months |
| Pisaniello et al, 2005, Italy | 120 | 42.3 | 67/53 (55.8%/44.2%) | 14 (11.6% | NA | 120ΤΤ (100%) | GroupA (A1-benign/A2-malignancy): Ca lactogluconate/ carbonate 300mg/day, GroupB (B1- benign /B2- malignancy): Ca carbonate 1.5gr + Cholecalciferol 400IU/day | 15 days postoperatively | < 8mg/dl | Early and combined oral administration of both calcium and vitamin D seemed to prove major efficacy in preventing and treating post-operative hypocalcemia. | 4 months |
| Lee et al, 2019, China | 134 | 50.1 **±** 11.9 | 117/17 (87.3%/12.7%) | 134 (100%) | 0 | 40TT (29.9%), 94 TT+CND (70.1%) | Intervention: 1500mg Ca/day + 1000IU/day Cholecalciferol, Controls: No treatment | 2 weeks postoperatively | ionized Ca < 4.6mg/dl | Routine supplementation with low dose calcium (1500mg/day) after thyroidectomy did not reduce the rate of symptomatic hypocalcemia or the need for iv calcium. POD 1 PTH level < 13 pg/mL could be a predictive factor for the development of postoperative hypocalcemia. | 6 months |
| Kurukahvecioglu et al, 2007, Turkey | 487 | 47.4 **±** 12 | 401/86 (82.3%/17.7%) | 76(15.6%) | NA | 487TT (100%) | Intervention: Ca 600mg/day + VitD 400IU/day, Controls: No treatment | 7 days postoperatively | symptoms that required Ca supplementation | Routine postoperative calcium and VitD supplementation may be useful for the prevention of symptomatic hypocalcemia after total thyroidectomy and may allow for a safe and early discharge. | NA |
| Li et al, 2022, China | 203 | 41 (19-78)* | 158/45 (77.8%/22.2%) | 203 (100%) | 8 (3.9%) | 147 TT+CND (72%)/ 57 TT+CND+LND (28%) | GroupA: No treatment, GroupB: 4.5gr / 6gr Calcium carbonate/day + 0.5μg Calcitriol/day | 1 month postoperatively | NA | Calcium and vitamin D supplementation administered exclusively to symptomatic patients achieved the same effect on protracted hypoparathyroidism as routine supplementation. However, routine supplementation significantly reduced postoperative hypocalcemia. | 6 months |
| Gkanis et al, 2025, Greece | 600 | 53.19 **±** 13.97 | 447/153 (74.5%/25.5%) | 184 (30.7) | 36 (6%) | 600TT (100%) | Intervention: 3gr Ca carbonate/gluconate + 2 mcg vitamin D (Alfacalcidol)/ day for 5 days and 2gr Ca carbonate/gluconate + 1 mcg Alfacalcidol for another 10 days, Controls: No treatment | 15 days postoperatively | <8.5mg/dl | Statistical lower incidence of biochemical and clinical hypocalcemia as well as shorter hospital stay in the treatment group. | 6 months |
| Mercante et al, 2019, Italy | 169 | 54 (21-82)* | 120/49 (71.0%/29.0%) | 71 (42%) | NA | 132TT (78.1%)/ 19 TT+CND (11.2%)/ 18TT+CND+LND (10.7%) | GroupA: 2grCa + 1mg 1,25OH2VitD, GroupB: on demand, Group C: if iPTH<10pg/dl | 4 days postoperatively | TSCa<2mmol/L, ICa< 1mmol/L | The preventive strategy was the most cost-effective even if some patients received an overtreatment of routine oral calcium and vitamin D supplementation for 4 days. | NA |
| Hao et al, 2021, China | 182 | 41.5 **±** 10.7 | 56/126 (30.8%/69.2%) | 182 (100%) | 0 | 182TT+CND (100%) | (Cohort 1) : GroupA: 1800mg Ca carbonate, GroupB: 1800mg Ca carbonate + 0.5μg Calcitriol / (Cohort 2): GroupC 1800mg Ca carbonate, GroupD: 1800mg Ca carbonate + 0.5μg Calcitriol | 3 days postoperatively | <8.4mg/dl | RDP-based postoperative management may reduce the risk of clinical hypocalcemia after thyroidectomy. Calcitriol supplementation appears necessary in patients with an RDP >70%, whereas it is not required when RDP is ≤70%. | 12 months |
| Jaan et al, 2017, India | 60 | 37.3 **±** 13.8 | 46/14 (76.7%/23.3%) | 45 (75%) | 1 (1.7%) | 52 TT (86.7%)/ 8 TT +LND (13.3%) | Group1: Ca 2 gr/day + Calcitriol 1mcg/day, Group2: No treatment | 7 days preop and 7 days postoperatively | <8.5mg/dl | Routine pre and post TT calcium and Vitamin D supplementation can significantly reduce post operative hypocalcemia and decrease prolonged hospitalization of patients and costs associated with multiple blood sampling. | 1 month |
| Nemade et al, 2012, India | 48 | 45.5 (26-65)* | 47/1 (97.9%/2.1%) | 7 (14.6%) | NA | 41TT (85.4%), 7TT+MRND (14.6%) | GroupA: Ca carbonate 2gr/day, Group B: Ca 2gr/day + VitD 60000IU three times a week | 1 week preop and 2 weeks postoperatively | <8mg/dl | Perioperative Ca + VitD supplement prevented a significant decrease of calcium levels and the subsequent development of major symptoms. | 6 months |
| Ghafouri et al, 2014, Iran | 43 | 40.9 **±** 11 | 32/11 (74.4%/25.6%) | 9 (20.9%) | 4 (9.3%) | 20STT (46.5%), 14TT (32.6%),9TT+CND (20.9%) | Intervention: 2gr Ca carbonate 12 hours before and 3gr/day Ca carbonate postoperatively, Controls: No treatment | 12 hours preop - 7 days postoperatively | NA | Prophylactic oral calcium supplementation decreases the incidence of symptomatic hypocalcemia and the need for calcium infusion 24 hours after total or subtotal thyroidectomy. It may also allow patient discharge from the hospital earlier. | 1 week |
| El-Shinawi et al, 2014, Egypt | 50 | 37.7 **±** 10 | 41/9 (82.0%/18.0%) | 7 (14%) | 0 | 50TT (100%) | Intervention: 1.5gr/day Ca (Calcimate)+ Alfacalcidol 0.5μg/day, Controls: No treatment | 4 weeks postoperatively | <8.4mg/dl | The hypocalcemic symptoms were minimal in the treatment group and more severe in the control group not receiving the supplement. Serum calcium levels decreased in both groups after surgery but were less in the treatment group. | 2 months |
| Li_Tian et al, 2022, China | 172 | 44.97 **±** 10.74 | 126/46 (73.3%/26.7%) | 172 (100%) | 0 | 130TT +CND (75.6%), 42TT+CND+MRND (24.4%) | Group A: Calcitriol 0.25μg/day + 1200mg Ca carbonate/day, Group B: 1200mg Ca carbonate, Group C: No treatment | 2 days preop and 1 day postoperatively | <8.44mg/dl | A short preoperative course of oral calcitriol and calcium carbonate for patients undergoing total thyroidectomy and bilateral CND reduces both the incidence of symptomatic and biochemical hypocalcemia, especially for those with transient hypoparathyroidism. | 6 months |
| * Values are presented as median (range); *TT*: Total Thyroidectomy; *Ca*: Calcium; *POD*: Postoperative Day; *MRND*: Modified Radical Neck Dissection; *CND*: Central Neck Dissection; *PTH*: Parathormone Hormone; *NA*: Not Available; *VitD*: Vitamin D; *LND*: Lateral Neck Dissection; *TSCa*: Total Serum Calcium; *Ica*: Ionized Calcium; *RDP*: Relative Decline of PTH; *preop*: preoperatively; *STT*: Subtotal Thyroidectomy | | | | | | | | | | | |

**Supplementary Table S2.** Sensitivity analysis excluding trials judged as overall high risk of bias according to the RoB 2 assessment.

| **Outcome** | **Comparison** | **Main analysis OR (95% CrI)** | **Sensitivity analysis OR (95% CrI)** |
| --- | --- | --- | --- |
| **Clinical hypocalcemia** | No treatment vs Calcium + VitD | 0.31 (0.17–0.51) | 0.31 (0.14–0.58) |
|  | Calcium vs Calcium + VitD | 0.52 (0.25–1.04) | 0.54 (0.20–1.42) |
|  | No treatment vs Calcium | 0.60 (0.26–1.26) | 0.57 (0.20–1.47) |
| **Biochemical hypocalcemia** | No treatment vs Calcium + VitD | 0.27 (0.17–0.42) | 0.30 (0.18–0.48) |
|  | Calcium vs Calcium + VitD | 0.44 (0.22–0.83) | 0.61 (0.26–1.36) |
|  | No treatment vs Calcium | 0.62 (0.33–1.19) | 0.49 (0.22-1.12) |
| **Intravenous calcium requirement** | No treatment vs Calcium + VitD | 0.15 (0.05–0.32) | 0.14 (0.03–0.36) |
|  | Calcium vs Calcium + VitD | 0.36 (0.10–1.00) | 0.50 (0.12–2.26) |
|  | No treatment vs Calcium | 0.43 (0.12–1.17) | 0.28 (0.05–0.92) |

Odds ratios (OR) and 95% credible intervals (CrI) from the main network meta-analysis are compared with estimates obtained after exclusion of studies judged as overall high risk of bias. The direction and magnitude of treatment effects remained largely unchanged across all outcomes.

*OR*: odds ratio; *CrI*: credible interval; *VitD*: vitamin D

**Supplementary Table S3.** Sensitivity analysis excluding studies using native Vitamin D preparations.

| **Outcome** | **Comparison** | **Main analysis OR (95% CrI)** | **Sensitivity analysis OR (95% CrI)** |
| --- | --- | --- | --- |
| **Clinical hypocalcemia** | No treatment vs Calcium + VitD | 0.31 (0.17–0.51) | 0.31 (0.18–0.45) |
|  | Calcium vs Calcium + VitD | 0.52 (0.25–1.04) | 0.58 (0.33–1.05) |
|  | No treatment vs Calcium | 0.60 (0.26–1.26) | 0.53 (0.26–0.92) |
| **Biochemical hypocalcemia** | No treatment vs Calcium + VitD | 0.27 (0.17–0.42) | 0.28 (0.18–0.43) |
|  | Calcium vs Calcium + VitD | 0.44 (0.22–0.83) | 0.50 (0.26–0.99) |
|  | No treatment vs Calcium | 0.62 (0.33–1.19) | 0.56 (0.29–1.06) |
| **Intravenous calcium requirement** | No treatment vs Calcium + VitD | 0.15 (0.05–0.32) | 0.16 (0.05–0.33) |
|  | Calcium vs Calcium + VitD | 0.36 (0.10–1.00) | 0.37 (0.11–0.96) |
|  | No treatment vs Calcium | 0.43 (0.12–1.17) | 0.44 (0.13–1.10) |

Odds ratios (OR) and 95% credible intervals (CrI) from the main network meta-analysis are compared with estimates obtained after exclusion of studies using native Vitamin D preparations. The direction and magnitude of treatment effects remained largely unchanged across all outcomes.

*OR*: odds ratio; *CrI*: credible interval; *VitD*: vitamin D

**Supplementary Table S4**. Raw study-level data used for the network meta-analysis.

| **Study** | **Treatment arm** | **Total n** | **Clinical hypocalcemia events** | **Biochemical hypocalcemia events** | **IV calcium events** | **Length of hospital stay (mean ± SD)** |
| --- | --- | --- | --- | --- | --- | --- |
| Langner_2016 | Calcium | 20 | NR | 6 | NR | NR |
| Langner_2016 | No_Treatment | 27 | NR | 17 | NR | NR |
| Roh_2006 | Calcium_VitD | 45 | 3 | 6 | 0 | NR |
| Roh_2006 | No_Treatment | 45 | 11 | 16 | 5 | NR |
| Roh_2009 | Calcium_VitD | 49 | 1 | 4 | 0 | NR |
| Roh_2009 | Calcium | 49 | 6 | 12 | 2 | NR |
| Roh_2009 | No_Treatment | 50 | 13 | 22 | 7 | NR |
| Ravikumar_2017 | No_Treatment | 52 | NR | 30 | 14 | NR |
| Ravikumar_2017 | Calcium | 52 | NR | 26 | 12 | NR |
| Ravikumar_2017 | Calcium_VitD | 104 | NR | 16 | 5 | NR |
| Bellantone_2002 | No_Treatment | 46 | 11 | NR | 2 | NR |
| Bellantone_2002 | Calcium | 49 | 2 | NR | 0 | NR |
| Bellantone_2002 | Calcium_VitD | 52 | 3 | NR | 0 | NR |
| Arer_2016 | Calcium_VitD | 53 | 1 | NR | 1 | NR |
| Arer_2016 | No_Treatment | 53 | 18 | NR | 0 | NR |
| Choe_2010 | Calcium_VitD | 154 | 36 | 31 | 16 | NR |
| Choe_2010 | No_Treatment | 150 | 65 | 50 | 27 | NR |
| Tartaglia_2005 | Calcium_VitD | 215 | 52 | NR | NR | NR |
| Tartaglia_2005 | Calcium | 202 | 60 | NR | NR | NR |
| Pisaniello_2005 | Calcium | 60 | 4 | NR | 0 | NR |
| Pisaniello_2005 | Calcium_VitD | 60 | 1 | NR | 1 | NR |
| Lee_2019 | Calcium_VitD | 65 | 21 | NR | NR | NR |
| Lee_2019 | No_Treatment | 69 | 15 | NR | NR | NR |
| Kurukahvecioglu_2007 | Calcium_VitD | 243 | 10 | NR | 0 | NR |
| Kurukahvecioglu_2007 | No_Treatment | 244 | 11 | NR | 7 | NR |
| Li_2022 | No_Treatment | 99 | 42 | 11 | NR | NR |
| Li_2022 | Calcium_VitD | 104 | 28 | 6 | NR | NR |
| Gkanis_2025 | Calcium_VitD | 300 | 17 | 51 | 6 | 1.25 ± 0.58 |
| Gkanis_2025 | No_Treatment | 300 | 37 | 120 | 28 | 1.7 ± 1.2 |
| Mercante_2019 | Calcium_VitD | 56 | 7 | 4 | 4 | 3.66 ± 1.16 |
| Mercante_2019 | No_Treatment | 57 | 12 | 19 | 6 | 3.89 ± 1.42 |
| Hao_2021 | Calcium | 91 | 30 | NR | 30 | NR |
| Hao_2021 | Calcium_VitD | 91 | 9 | NR | 16 | NR |
| Jaan_2017 | Calcium_VitD | 30 | 3 | 6 | 0 | NR |
| Jaan_2017 | No_Treatment | 30 | 12 | 13 | 4 | NR |
| Nemade_2012 | Calcium | 24 | 10 | 7 | 3 | NR |
| Nemade_2012 | Calcium_VitD | 24 | 3 | 1 | 0 | NR |
| Ghafouri_2014 | Calcium | 23 | 1 | NR | 2 | 2.1 ± 0.3 |
| Ghafouri_2014 | No_Treatment | 20 | 6 | NR | 10 | 2.9 ± 1.4 |
| El-Shinawi_2014 | Calcium_VitD | 25 | 2 | 2 | 0 | 2.28 ± 0.61 |
| El-Shinawi_2014 | No_Treatment | 25 | 6 | 3 | 6 | 2.88 ± 1.54 |
| Li_Tian_2022 | Calcium_VitD | 63 | 16 | 24 | 3 | NR |
| Li_Tian_2022 | Calcium | 52 | 22 | 21 | 7 | NR |
| Li_Tian_2022 | No_Treatment | 57 | 25 | 31 | 10 | NR |

*IV:* Intravenous; *SD:* Standard Deviation; *NR:* Not Reported

**Supplementary Figures**


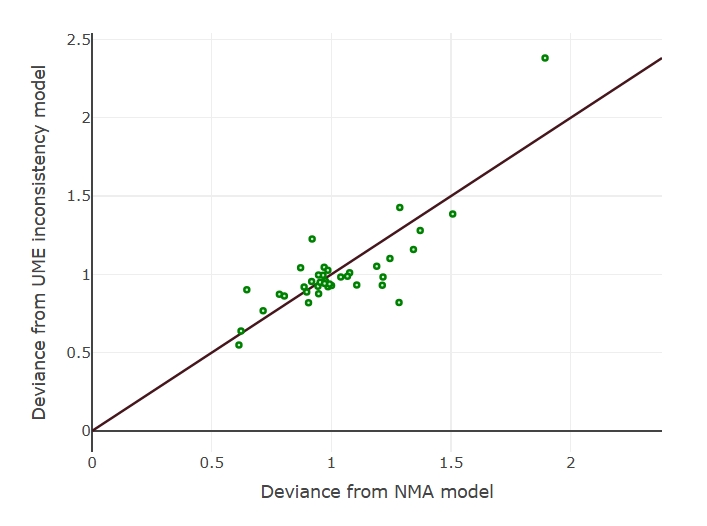


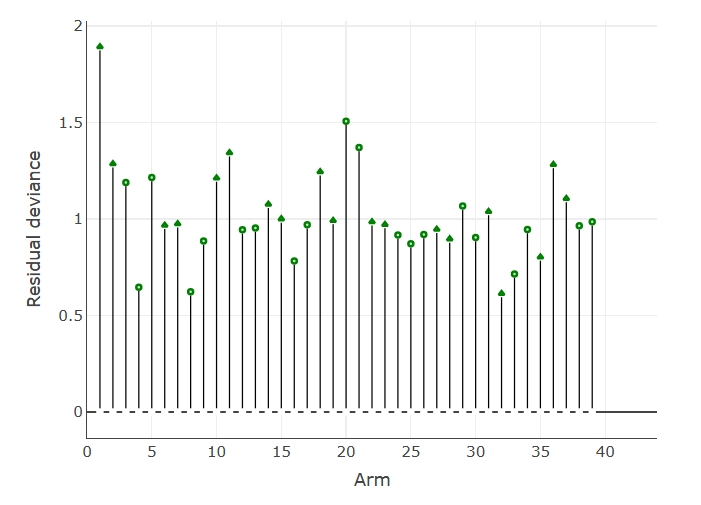


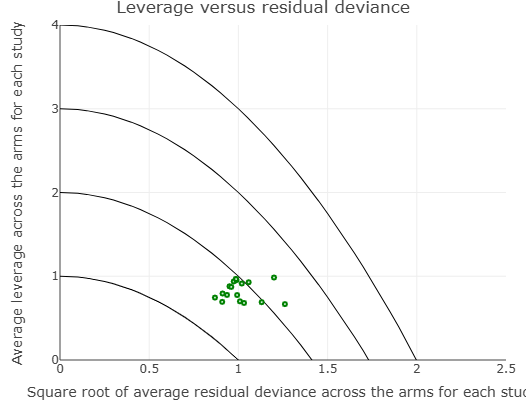


(C)

(A)

(B)

**Supplementary Figure S1**. Model diagnostics for the Bayesian network meta-analysis. (A) Deviance comparison between the consistency (NMA) and inconsistency (UME) models; alignment of points around the line of equality indicates good agreement between models and absence of important inconsistency. (B) per-arm residual deviance plot; values close to 1 suggest adequate model fit for individual data points. (C) Leverage versus residual deviance plot used to identify influential observations; absence of extreme leverage values indicates that no individual study exerted disproportionate influence on the model estimates**.**


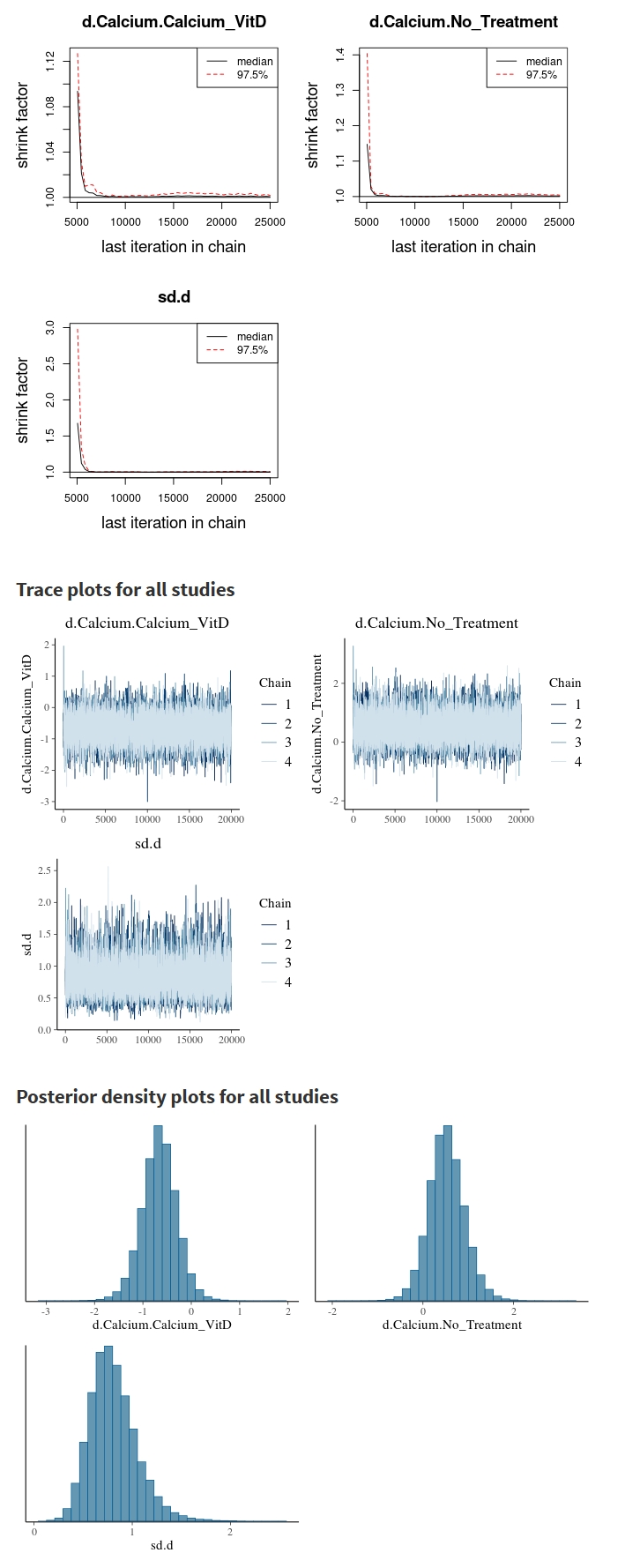


(B))

(A)

(C)

**Supplementary Figure S2**. Convergence diagnostics for the Bayesian network meta-analysis. (A) Gelman–Rubin shrink factor plots showing that potential scale reduction factors approached 1.00 for all parameters, indicating adequate convergence of the Markov chains. (B) Trace plots for four Markov chains demonstrating good chain mixing across iterations. (C) Posterior density plots illustrating stable and unimodal posterior distributions of treatment effects. *“sd.d”* denotes the between-study standard deviation (heterogeneity parameter) of the random-effects model.
